# Spatial mapping of metals in tissue-sections using combination of mass-spectrometry and histology through image registration

**DOI:** 10.1038/srep40169

**Published:** 2017-01-10

**Authors:** Jiri Anyz, Lenka Vyslouzilova, Tomas Vaculovic, Michaela Tvrdonova, Viktor Kanicky, Hajo Haase, Vratislav Horak, Olga Stepankova, Zbynek Heger, Vojtech Adam

**Affiliations:** 1 Department of Cybernetics, Faculty of Electrical Engineering, Czech Technical University, Technicka 2, CZ-166 27 Prague 6, Czech Republic; 2 Czech Institue of Informatics, Robotics, and Cybernetics, CTU in Prague, Jugoslávských partyzánů 1580/3, CZ-160 00 Prague 6, Czech Republic; 3 Department of Chemistry, Faculty of Science, Masaryk University, Kotlarska 2, CZ-611 37 Brno, Czech Republic; 4 Central European Institute of Technology, Masaryk University, Kamenice 5, CZ-625 00 Brno, Czech Republic; 5 Department of Food Chemistry and Toxicology, Berlin Institute of Technology, Gustav-Meyer-Allee 25, D-133 55 Berlin, Germany; 6 Laboratory of Tumour Biology, Institute of Animal Physiology and Genetics, Academy of Sciences of the Czech Republic, v.v.i., Rumburska 89, CZ-277 21 Libechov, Czech Republic; 7 Department of Chemistry and Biochemistry, Mendel University in Brno, Zemedelska 1, CZ-613 00 Brno, Czech Republic; 8 Central European Institute of Technology, Brno University of Technology, Technicka 3058/10, CZ-616 00 Brno, Czech Republic

## Abstract

We describe a new procedure for the parallel mapping of selected metals in histologically characterized tissue samples. Mapping is achieved via image registration of digital data obtained from two neighbouring cryosections by scanning the first as a histological sample and subjecting the second to laser ablation inductively coupled plasma mass spectrometry. This computer supported procedure enables determination of the distribution and content of metals of interest directly in the chosen histological zones and represents a substantial improvement over the standard approach, which determines these values in tissue homogenates or whole tissue sections. The potential of the described procedure was demonstrated in a pilot study that analysed Zn and Cu levels in successive development stages of pig melanoma tissue using MeLiM (*Me*lanoma-bearing-*Li*bechov-*M*inipig) model. We anticipate that the procedure could be useful for a complex understanding of the role that the spatial distribution of metals plays within tissues affected by pathological states including cancer.

Intensive multidisciplinary cooperation involving the life sciences and technology has produced complete genomic DNA sequences for a number of living organisms, but the differences between normal and pathological cellular functions remain to be fully elucidated. Zn, the most abundant transition metal in most cells, plays a vital role in the functions of more than 300 enzymes, DNA stabilization, and the regulation of gene and protein expression. Zn modulates various biological processes, including apoptosis^1^, homeostasis, and oxidative stress^2^, and multiple functions of the immune system^3^.

Zn is also an important player in various diseases, including cancer and aging-related disorders. One of the largest meta-analyses (22,737 participants) of the role of Zn in cancer yielded somewhat conflicting results: elevated serum Zn levels were not observed in any type of cancer, and decreased serum Zn levels were observed only in lung, head and neck, breast, liver, stomach, and prostate cancers^4^. However, Zn patterns were evident at the tissue level, with increased levels in breast cancer tissue and decreased levels in prostate, liver, lung, and thyroid cancers. Ambiguous results for serum and tissue Zn levels were reported for the other included tumours across the analysed studies. These findings indicate the complex role that Zn plays during the transformation of normal tissue to cancerous one.

The observed range of Zn values suggests that Zn levels are influenced by numerous factors, including the origin of the studied tissue, particularly the structural characteristics of the tissue. Cancerous tissue is histologically heterogeneous; cancer cells are unevenly dispersed and form clusters of different sizes that are surrounded by normal stromal cells. To elucidate the underlying processes, data must be obtained from histologically homogeneous parts of the studied cancer sample. New methods that can integrate this additional information are needed. We have therefore designed a method that automates the process of combining and matching spatially resolved results from diverse imaging techniques, namely, histological and spectroscopic descriptions of the studied samples. We validated this new method in a case study whereby the Zn and Cu contents and their distribution in pig melanoma using the MeLiM (*Me*lanoma-bearing *Li*bechov *M*inipig) model were analysed. In cancer-affected MeLiM animals, spontaneous regression of melanoma and rebuilding into fibrous tissue frequently occur during postnatal development^5^. The proposed method was used to detect and quantify both the Zn and Cu biometals directly in very small, histologically uniform and mutually distinct zones of melanoma samples, providing an efficient method for monitoring changes in metal levels during the process of spontaneous regression. The same method can be easily modified for applications to other biometals and/or tissue samples.

## Results

### Histological heterogeneity of pig melanoma in the MeLiM model

**Figure 1:**
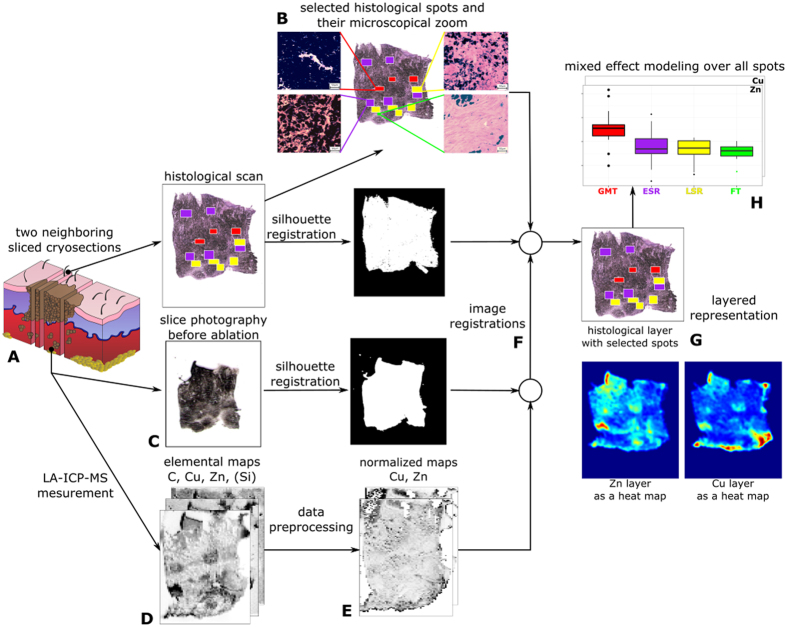
A schematic description of the whole transformation process from cryosections to matched layers representation and statistical evaluation. (**A**) The two cryosections were sliced from tissue, (**B**) the narrow slice is used for histological analysis and to find uniform spots - red - GMT (normally growing melanoma tissue), violet - ESR (early spontaneous regression), yellow - LSR (late spontaneous regression) and green - FT (fibrous tissue). (**C**) The wide slice is photographed and (**D**) measured by LA-ICP-MS. (**E**) The measured elemental maps are normalized with respect to dry weight. The slices are registered in two steps. (**F**) Firstly, the normalized metal maps are registered with the slice photography and secondary, the result of the first step is registered with the histological scan. The registration is based on silhouette registration. (**G**) The output of the process is a layered representation of tissue consisting of a histological layer with selected spots and metal layers. (**H**) The layered representation of all tissues is statistically evaluated. Our data confirm the hypothesis that content of zinc in the zone of growing melanoma tissue (GMT) is significantly greater than in all remaining zones.

**Figure 2:**
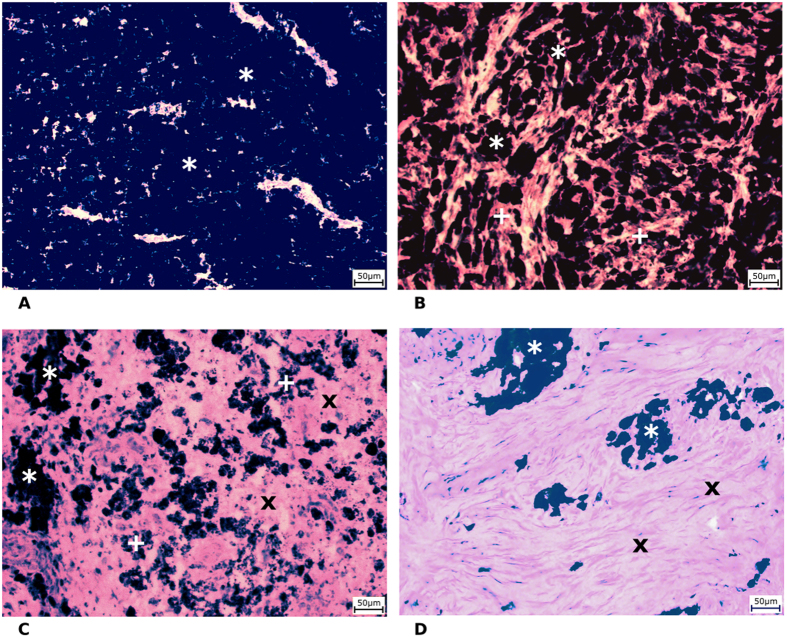
Four histologically differing zones identified in haematoxylin-eosin stained skin porcine melanoma: (**A**) growing melanoma tissue (GMT), (**B**) melanoma tissue with early destruction of melanoma cells (early spontaneous regression-ESR), (**C**) melanoma tissue with late destruction of melanoma cells (late spontaneous regression-LSR), (**D**) fibrous tissue (FT) with a few remaining melanoma cells. Scale bar = 50 μm (* = melanoma cells; + = cellular debris from damaged melanoma cells; x = fibrous tissue).

The MeLiM (*Me*lanoma-bearing *Li*bechov *Mi*nipig) strain of miniature pigs with heritable cutaneous melanoma is an original animal cancer model with histopathological, biochemical and molecular biological similarities to human melanoma^6^,^7^,^8^,^9^. Multiple skin melanomas appear at birth or shortly thereafter in approximately half of all piglets. More than 2/3 of the affected minipigs display complete spontaneous regression of tumours, which is usually accompanied by skin and bristle depigmentation. After a short postnatal period of tumour growth, the first signs of spontaneous regression, both macroscopic (flattening and grey colour of tumours) and microscopic (gradual destruction of melanoma cells, reduced expression of collagen IV and laminin, and rebuilding of tumour tissue into fibrous tissue), are observed. Ten weeks of age appears be a turning point in the transition between tumour growth and spontaneous regression in MeLiM melanoma^5^. The incidence of spontaneous regression of melanoma is high in the MeLiM model. Melanoma cells are gradually destroyed after a short postnatal period of tumour growth, and the tumour tissue is rebuilt into fibrous tissue. In connection with this process, four structurally different zones were distinguished in the histological samples and marked with various colours (Fig. 1): GMT (the zone of normally growing melanoma tissue - red rectangles), ESR (the zone of early spontaneous regression - violet rectangles), LSR (the zone of late spontaneous regression - yellow rectangles) and FT (the zone of fibrous tissue – green rectangles). A detailed histological view of the zones and their description are given in Fig. 2A–D. Particularly, using haematoxylin-eosin staining, four histologically different zones were distinguished in the collected melanoma samples (Fig. 2A–D):

- The zone of normally growing melanoma tissue (GMT) was composed of heavily pigmented, intact melanoma cells, which were distributed close together with narrow extracellular spaces (Fig. 2A).
- The zone of early melanoma cell destruction (early spontaneous regression, ESR) included cellular debris from some of the damaged melanoma cells, but a considerable number of melanoma cells were still well preserved (Fig. 2B).
- The zone of late melanoma cell destruction (late spontaneous regression, LSR) was characterized by extensive damage to the melanoma tissue (forming predominantly cellular debris with small groups or individually dispersed melanoma cells) and its incipient rebuilding in the fibrous tissue (Fig. 2C).
- The zone of fibrous tissue (FT) arising by the total rebuilding of tumour tissue. A small number of remaining melanoma cells were occasionally still present (Fig. 2D).

Age-dependent changes in melanoma structure were clearly observed. In the melanoma of the youngest (4-week-old) animals, zones of normally growing melanoma tissue were distinctly prevalent compared with zones of early melanoma cell destruction. The other two zones were entirely missing. The number and size of the GMT zones decreased with age, whereas the opposite tendency was observed in the zones of ESR and late melanoma cell destruction (the latter appeared in 6-week-old animals). The damaged tumour tissue was gradually replaced by fibrous tissue, which was first observed in 15-week-old minipigs. In the melanoma of the oldest animals (22 weeks old), zones of late destruction of melanoma cells were most prevalent, and fibrous tissue occupied the areas between the zones. In these minipigs as well as in the 15-week-old minipigs, zones of GMT were no longer observed. Using the method suggested in the present paper, selected zones were matched with the elemental map (of the neighbouring cryosection) as provided by laser ablation with the aim of comparing Zn and Cu content in melanoma during melanoma growth and successive stages of spontaneous regression.

### Elemental imaging

Laser ablation inductively coupled plasma mass spectrometry (LA-ICP-MS) enables the measurement of the metal content in a selected zone ranging from one to several hundred micrometres on the sample surface. LA-ICP-MS provides an ideal, rich source of information because it can match each ablation pixel (the smallest part of the studied sample that can be distinguished using ablation) to the relevant quantified information about the presence of most chemical elements. Laser ablation parameters, such as laser beam fluence, laser spot size, and scan speed rate, determine the time necessary for analysis of any sample as well as the accuracy of the obtained results. These parameters were optimized to ensure the required performance, namely a low limit of detection (LOD) and low broadening of images within a reasonable time period of analysis. This optimization was performed by ablation of white paper with printed ink lines of 800 μm thickness. For this purpose, the ^63^Cu signal was recorded because this element is present in the used ink^10^.

Elemental mapping was performed using line scan mode so that each line started on a glass substrate outside the tumour tissue. The laser beam was moved on the sample surface continuously along a straight line with a constant scan rate of 200 μm/s. The laser beam diameter and the distance between individual straight lines were both 100 μm. The laser beam fluence and the repetition rate were optimized, and respective values of 8 J/cm^2^ and 20 Hz were used for all LA-ICP-MS analyses. The high laser beam fluence was used to prevent influence of different ablation rates (see section “*2D imaging*” for more details) and was optimized to reach the glass substrate during laser ablation.

**Figure 3:**
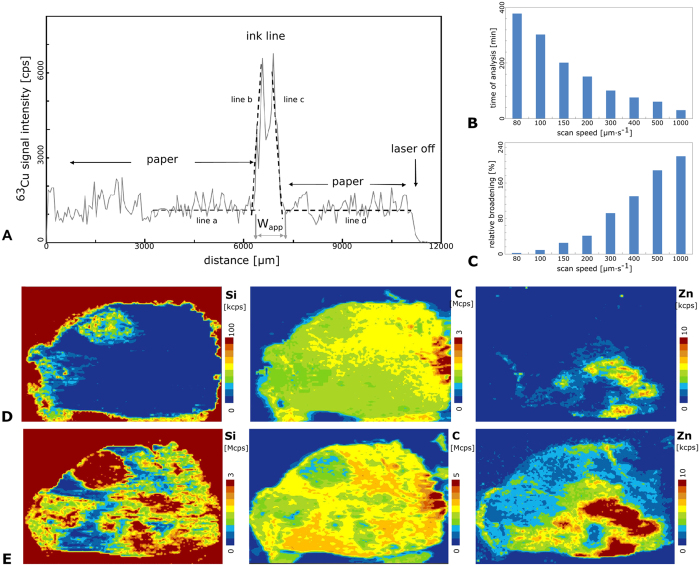
(**A**) LA-ICP-MS signal in line scan mode recorded for laser beam pass across one printed line at laser spot diameter of 100 μm, scan speed of 20 μm s^−1^, laser beam fluence of 8 J cm^−2^ and repetition rate of 10 Hz. (**B**) Duration of scanning of the sample area of 15 × 15 mm at various scan speeds (μm s^−1^). (**C**) Relative broadening of a printed line (expressed in %) with width of 800 μm obtained at various scan speeds (μm s^−1^). (**D**) Elemental maps of C, Si and Zn obtained at “soft” ablation parameters (2 J/cm^−2^) for tissue K320/1 (12 weeks old). (**E**) Elemental maps of C, Si and Zn obtained at “hard” ablation parameters (8 J/cm^−2^) for tissue K320/1 (12 weeks old).

The quantification was based on calibration performed using agarose gel standards prepared by spiking with known amounts of Cu and Zn, for Cu see in Fig. 3A. The prepared calibration standards contained single metal content of 0, 20, 100, 500 and 2000 mg/kg. Each standard was ablated in triplicate using the same ablation parameters used for imaging. Background correction was performed by subtraction of the average signal obtained using a carrier gas blank (He).

### Ablation parameter setting and relative broadening of images

In imaging by means of LA-ICP-MS, a broadening of imaged patterns occurs that can be evaluated as described previously^10^,^11^,^12^. The broadening is mainly due to a combination of the laser spot size and scan speed rate. Hence, these parameters must be carefully adjusted with respect to the size of the treated samples (due to time of analysis) and the size of the zones of interest (due to trueness of imaging).

Histologically different zones in minipig tumour tissues were well-defined areas inside the analysed tissue samples with a size of several hundred micrometres in each dimension. Eight scan speed rates were used for the optimization (80, 100, 150, 200, 300, 400, 500 and 1000 μm/s). Due to the large dimensions of the imaged tissue samples (approximately 8 × 5 mm), a laser spot size of 100 μm was selected to reach a minimal LOD. An increase in the laser spot size resulted in a lower LOD^10^,^13^. The apparent width **w**_**app**_ was calculated to evaluate the broadening caused by the various scan speed rates. **w**_**app**_ was obtained as the difference between the onset of the signal increase and the end of its decrease after the laser spot passed across the testing pattern (ink line). The onset points were obtained as the intersections of the trend lines **a** and **b**, and the endpoints were obtained as the intersections of the trend lines c and d. The trend lines **a** and **d** were obtained from linear regression fitted to the domains of the signal between the printed lines, whereas the trend lines 2 and 3 were obtained by linear regression in the domains of signal rise and drop, respectively. The trueness of imaging was expressed as the relative broadening **Δw**_**rel**_ of the image **w**_**app**_ of the printed line with respect to its real width **w**.

The dependence of the relative broadening on the scan speed rate is displayed in Fig. 3C. The relative broadening increased from 5% to more than 200% as the scan speed rate increased from 80 to 1000 μm/s.

However, the lateral resolution and LOD were not the only parameters considered in developing the LA-ICP-MS elemental mapping method. The duration of analysis is an important parameter because it affects the operating costs. The times required for mapping are displayed in Fig. 3B and were calculated for typical thin sections of our samples of tumour tissue (8 × 5 mm). The time required for analysis decreased with the increasing scan speed rate: whereas approximately 400 minutes was needed for a scan speed rate of 80 μm/s, only 20 minutes was needed for a scan speed rate of 200 μm/s. However, the broadening observed for these parameters was greater than 200%. Hence, a scan speed rate of 200 μm/s was selected as an optimal compromise because it resulted in a relative broadening of 40% and duration of analysis of 150 min.

### 2D imaging

Laser beam fluence is one of the most crucial parameters for laser ablation. The laser beam fluence mainly affects the ablation rate, the amount of material released during one laser pulse. Variations of the ablation rate complicate the quantification of LA-ICP-MS experiments because each laser pulse releases different amounts of analysed material in the selected range. There are multiple methods to compensate for this uncertainty. The first approach utilizes normalization to the sum of 100%^14^,^15^ and can be successfully used for single-spot analysis or imaging of materials with well-known matrix composition to determine the appropriate multiplication coefficient that results in the whole content of 100%. This approach cannot be used for samples with a complex matrix containing large amounts of non-determinable elements or their groups (e.g., fluoroapatite, in which OH^−^ is substituted by F^−^, or biological samples containing O, N and H).

In our case, the analysed tumour tissue represents samples containing large amounts of non-determinable elements (O, N, and H). Hence, the normalization approach based on the sum of 100% cannot be used successfully. The second normalization approach is based on utilization of an internal standard^16^, i.e., monitoring an isotope with a known amount. It is necessary to rely on internal standards such as C, which is abundant in the sample. However, when carbon is used as an internal standard, marked systematic error arises due to the production of carbon-containing gaseous species, resulting in high losses of the carbon signal during laser ablation^17^.

As mentioned above, differences in the ablation rate complicate imaging due to variations in the amount of ablated tissue. This phenomenon could be minimized if controlled amounts of material were released during each laser pulse. Hence, we suggest a total mass removal approach when the whole layer of tissue is completely released. The Si signal indicates when the whole layer of tissue is removed and the glass substrate is ablated as well. Moreover, if the glass substrate does not contain special-interest elements (Zn, Cu and C), there is no danger of contamination from the glass substrate, and the signals of Zn, Cu and C arise from the tissue only.

Elemental images of two nearby thin sections were compared. One section was ablated at high laser beam fluence (8 J/cm^2^–hard ablation), and the second section was ablated at low laser beam fluence (2 J/cm^2^–soft ablation), whereas thickness of the given sections were considered to be critical and, thus, were controlled by the slicing machine, where relative standard deviation did not exceed 5% and did not significantly influence further discussed observations. The terms hard and soft ablation are used in this text for explanation only. When soft ablation is applied, the signal of ^28^Si corresponding to the ablation of the glass substrate under the tissue is not strongly enhanced compared to the gas blank value (Fig. 3D). Thus, the laser beam fluence is not sufficient to ablate the whole layer of the tissue, and the glass substrate is not reached, with the exception of two small regions in the left part of the tissue. When hard ablation is applied, significantly higher intensities of ^28^Si are observed (Fig. 3E). The range of the ^28^Si scale is 30 times larger than that of the soft ablation image, indicating that the glass substrate was reached and that the tumour tissue was ablated completely. In the case of the Zn image, we can observe strong enrichment in the lower right corner of tissue. The strongly enhanced Zn signal does not originate from the glass substrate, as confirmed by comparison with the parts of the image where the glass substrate was analysed only (red part from Si image and blue part of carbon image). The Zn signal is clearly close to zero in all of these regions.

### Arguments supporting the need for registration

**Figure 4:**
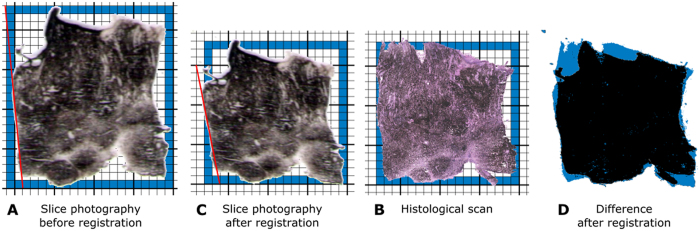
Photographs of two neighboring cryosections prepared to be subjected to laser ablation and to histological analysis (**A**,**B**). The image **C** is the result of registering the images (**A**,**B**). The blue rectangle indicates in the images (**A**–**C**) the minimal rectangle (with sides parallel to axes) the image fits in. The red line in the image (**A**,**C**) accentuates orientation of the corresponding borders on both the images. Images (**B**,**C**) are compared in the image (**D**): while the places appearing in both images have black color, the space in blue corresponds to symmetric difference of both images as explained in Supplementary Note 1.

As it was mentioned above, the data about the spatial distribution of each chemical element can be depicted in the form of an “element heat map” (see Fig. 3D,E). Because the heat maps of all elements can be obtained during one analysis of a single sample, the maps have identical shape, orientation and resolution and are thus ideal for addressing questions such as the relationship between the presence of Zn and Cu in selected ablation pixels of the studied sample. However, the task becomes much more complex when additional information, such as the histological properties of the considered zone, must be incorporated because such information must be determined from another tissue section. Two neighbouring serial cryosections of the original tissue sample must be available, one of which is subjected to ablation, whereas the other is subjected to standard staining for histological analysis. Both treatments produce digital images providing complementary information about the tissue sample. Data corresponding to the selected zone from both treatments must be paired. Fig. 4A and B present photographs of two neighbouring cryosections acquired under the same technical conditions; in an ideal case, the two samples should have identical contours. The two samples are intended for different types of treatment: the sample in Fig. 4A is ready to undergo the ablation procedure, whereas the sample in Fig. 4B will be subjected to a histological analysis. Both samples are so tender that handling them may change both their shape and orientation slightly; the tissue can stretch, or some of its parts may be pinched. Comparison of the two images reveals stretching in both dimensions, with a larger change in the vertical axis: the image in Fig. 4B fits into the blue rectangle with a size of 17.5 × 18, whereas an area of 20 × 23 is necessary for the image in Fig. 4A. Linear transformation of one of the images is suitable to solve this problem. Linear transformation of the image in Fig. 4A is followed by registration to match the histological scan of the sample in Fig. 4B. The resulting image, Fig. 4C (obtained by transformation of Fig. 4A), has a size of 18 × 18, which is very close to that of Fig. 4B. The area in which the two images do not match is clearly identified by blue colour in Fig. 4D.

Comparison of the slices before registration of Fig. 4A to B and after this registration (Fig. 4C) indicates that the slices do not have the same orientation. This can be clearly demonstrated through comparison of the angles between the red lines defining one of the borders in both pictures and the horizontal line. Let us estimate the corresponding tangent values using the scale underlying both images: while this value is 23/2 = 11.5 for the image **a**, it is 15/3 = 5 for the image **b**. The resulting difference in the orientation of both images is approximately 0.055π (or 10°).

Moreover, the size and granularity obtained from the heat map produced from LA-ICP-MS and the histological image can differ by an order of magnitude, depending on the applied magnification. Thus, the absolute size of a pixel in the histological image differs significantly from that of the ablation pixel. To make full use of the information about the spatial distribution of different metals in the sample, a homogeneous cluster of cells must be identified in the histological image, and the corresponding zone must be located in all considered element heat maps. This task can be approximately resolved manually by taking advantage of the human ability to match similar objects, as demonstrated in a breakthrough study of nine samples of invasive breast carcinoma^18^. However, manual matching is not a viable solution for frequent analysis of large sample sets. We therefore designed and developed a software method that automates the process of combining and matching spatially resolved results from diverse imaging techniques, namely histological and spectroscopic descriptions. Our method, schematized in Fig. 1, first registers the digital images to project the contours specified in one image to the corresponding pixels of the other images. Consequently, if a zone of interest Z is outlined in one of the images (e.g., histological image), the matching zone Z′ is identified automatically in any other image. Thus, it is possible to combine available complementary data about both matching zones Z and Z′ (e.g., histology description of Z and Zn content obtained from Z′), a necessary step towards a modern methodology of analysis, interpretation and integration of biochemical data from diverse sources.

### Image registration and creation of layered multidisciplinary description

Each tissue sample was submitted to analysis by two fully independent methods, namely histological scanning and the LA-ICP-MS measurement, and the results for the presence of Zn and Cu in specified histologically uniform locations of the sample were compared. Each of the applied methods processes (and destroys) one of the two bordering serial tissue sections from the same biological sample (Fig. 1A), whereas each delivers its results in the form of a digital image. Morphology of the studied tissue suggests that the corresponding zones in these sections can be assumed to represent identical histological structures provided the selected zones are placed inside of a histologically homogenous tissue and their diameter is several times bigger than the thickness of the used slices. These conditions were respected during data collection. A standard method for overlaying two different images that has been extensively studied in the context of computer vision and is referred to as image registration was applied^19^. Particularly, the methodology of image registration is well developed and offers ample approaches for overlaying two or more images of the same section obtained from different viewpoints or by different sensors^19^. The affine transformation^20^ was chosen for the registration of the studied images after considering other relevant methods. The main benefit of the affine transformation is its simplicity and understandability due to the linear transformation it applies to map the new image on the reference image. Let us assume that an image is a function of two variables ***I**(**x**, **y***) that assign an intensity value to the pixel with specific coordinates ***x*** and ***y***. The affine transformation of the 2D image is a simple linear mapping in the form of Equation No. 1:


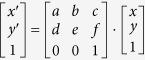


where ***x*** and ***y*** are the coordinates of pixels in the original image and ***x’*** and ***y’*** are the coordinates of the corresponding pixels in the transformed image. The treatment of the image is fully characterized by the constant parameters ***a-f*** of the 3 × 3 transformation matrix ***T*** in the middle of the equation. The affine transformation can accomplish translation, rotation, and scaling as well as shear deformation of pixels. The quality of the match between the reference image and the transformed image is characterized by a symmetric difference of both images, as depicted in Fig. 4. This difference should be zero in the ideal case.

Multiresolution image registration^21^ that applies an iterative gradient algorithm is one of the basic procedures for estimation of the parameters ***a-f*** of the transformation. It is robust and ensures good results. The registered images were reduced to silhouettes to simplify the parameter estimation and to avoid problems of different modalities of the registered images. The use of silhouettes allows for the definition of the brightness function ***I**(**x**, **y***), defined as follows:

*I(x, y*) = 1 *for a pixel belonging to the silhouette*

*I(x, y*) = 0 *elsewhere*

**Figure 5:**
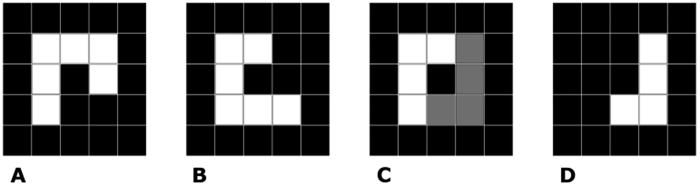
The illustration of the SSD criterion for image registration. The panels (A,B) show the reference image and the image to be registered. The panel (C) shows overlaying of both the images, the gray part corresponds to the difference between the images. The panel (D) shows the difference on itself. As the patterns are 3 × 3 pixels, the difference part corresponds to SSD of 4.

First, the registered image must be described in the same coordinate system as the reference image. In the next step, registration can be ensured. The sum of squared differences between the reference image and the registered image (SSD, Fig. 5) was chosen as the criterion to be minimized during the registration procedure. For the silhouettes of the reference image ***I***_***ref***_(***x**, **y***) and the image to be registered ***I***_***reg***_(***x**, **y***), the SSD may be defined by Equation No. 2:


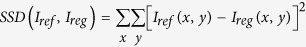


The parameters of the mapping between both images can be determined by minimizing the SSD with the gradient algorithm. To ensure convergence of the gradient algorithm, iterative (gradual) estimation of the parameters on a dyadic decomposition of the images was used [21]. This multiresolution image registration approach decomposes both considered images into a sequence of images with decreasing resolution (the resolution of each successive image is half that of the preceding image). The maximal length ***l***_***max***_ of the sequence of these decompositions depends on the integer part of the smallest dimension *d*_*min*_ (width and/or height “given in pixels of the considered image”) of both silhouettes and is given by the following expression as Equation No. 3:


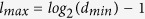


This upper limit for ***l***_***max***_ ensures that any of the images in the sequence will have at least 2 pixels in its smallest dimension. The procedure starts with the pair of images (reference and registered) with the lowest level of resolution. The parameters of the transformation are estimated for the given resolution by the gradient algorithm, and their values are used as the initial choice of parameters for the estimation of transformation in the next step, which treats the pair of images with resolution two times higher than the last (the iterative step). These steps are repeated until the original resolution of both images is reached and the final parameter estimates are obtained.

There are even more powerful types of transformations, but affine transformation proved to be sufficient for our purposes. All operations performed by the affine transformation on the image of the tissue sample may be identified with the actual treatment of the sample, such as compressing or stretching of the cryosection during cutting or shifting and rotating during the placement of the cryosection on the slide. The described transformation of coordinates must be followed by the interpolation of the original brightness function to obtain detailed information about the transformed image with respect to the new coordinate system. Linear interpolation was used in our case. Our aim is to provide complex information about individual areas of the tissue samples as provided by the considered methods for their analysis. The first step toward this goal is to determine the match between the histological scan and tissue slice photography and also between the tissue slice photography and the laser ablation measurements. The parameters of both transformations were estimated by MATLAB’s universal optimizer for unconstrained optimization supplied with the optimization toolbox.

### Output of the analysis with biological significance

**Figure 6:**
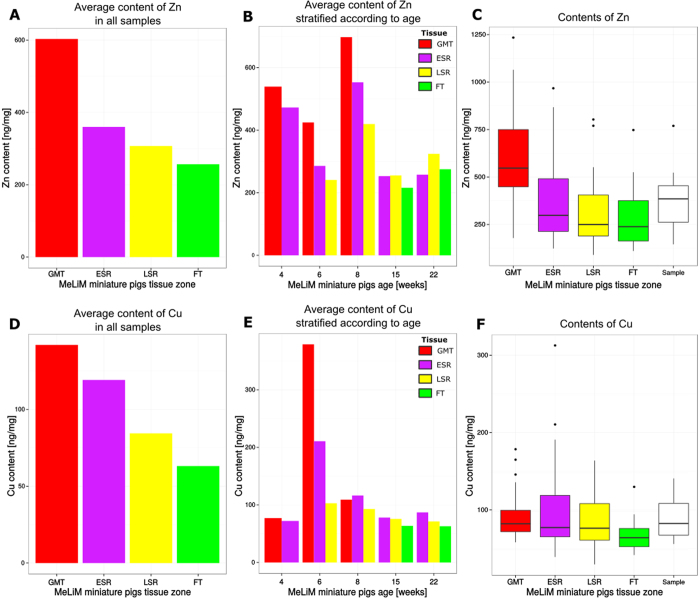
Average content of Zn and Cu in all samples (**A**,**D**) and in samples stratified according to age of minipigs (**B**,**E**). Box plots (**C**,**F**) visualize dispersion of data corresponding to average Zn and Cu content in specified tissue zones by samples as well as by individual samples as a whole for all 10 considered minipigs (the white box plot).

The preceding procedures provided data for 10 tissue samples each from 10 individual animals of five postnatal ages. Three histologically different zones were observed in each sample obtained from minipigs at 6 weeks of age or older (GMT, ESR and LSR at the age of 6 weeks; ESR, LSR and FT at the age of 15 and 22 weeks), two zones only (GMT and ESR) were detected in the samples from the 4-week-old minipigs. In each individual sample, 10 to 15 spots (3–5 per each zone) were annotated, resulting in 125 annotated spots that were subjected to the statistical analysis described in Supplementary Note 1. The sample means counted from all annotated spots, which were obtained by transforming the histological annotation into content map coordinates, revealed a tendency for decreasing Zn and Cu content during spontaneous disintegration of the melanoma tissue and rebuilding into fibrous tissue (see Fig. 6A–F and Supplementary Table 1). Because the individual animals had different overall metal contents (in the whole tissue sample), a linear mixed effect model^22^,^23^ was applied to distinguish differences originating from interindividual variation from actual differences among histologically different zones of tissue. Differences among the tissue zones were predicted as fixed effects, and the indicator of an animal was used as a random effect. The resulting ***p*** values estimated by non-parametric bootstrap test^24^ were corrected by the Bonferroni correction for multiple comparison^25^.

The exploration of values depicted in Supplementary Table 1 inspired four hypotheses on the presence of metals in different tissue zones. The testing of these hypotheses is documented in Supplementary Table 2. Table 1 reviews the estimated coefficients of the linear mixed effect model for two hypotheses: “The average Zn (or Cu) content in the GMT zones is lower than that in the remaining zones (ESR, LSR and FT).” To maintain the overall error rate at 0.05, the threshold for ***p*** values (used in the decision to refute the hypothesis) was 0.0125 according to the Bonferroni correction for multiple comparisons. The presented ***p*** values indicate that the null hypothesis can be rejected only in case of Zn. Our data confirm that the Zn content in the zone of growing melanoma tissue (GMT) was significantly greater than in all remaining zones, which represent consecutive stages of the tumour tissue that arise as a result of the spontaneous regression of melanoma (ESR, LSR) and its final rebuilding into fibrous tissue.

## Discussion

**Figure 7:**
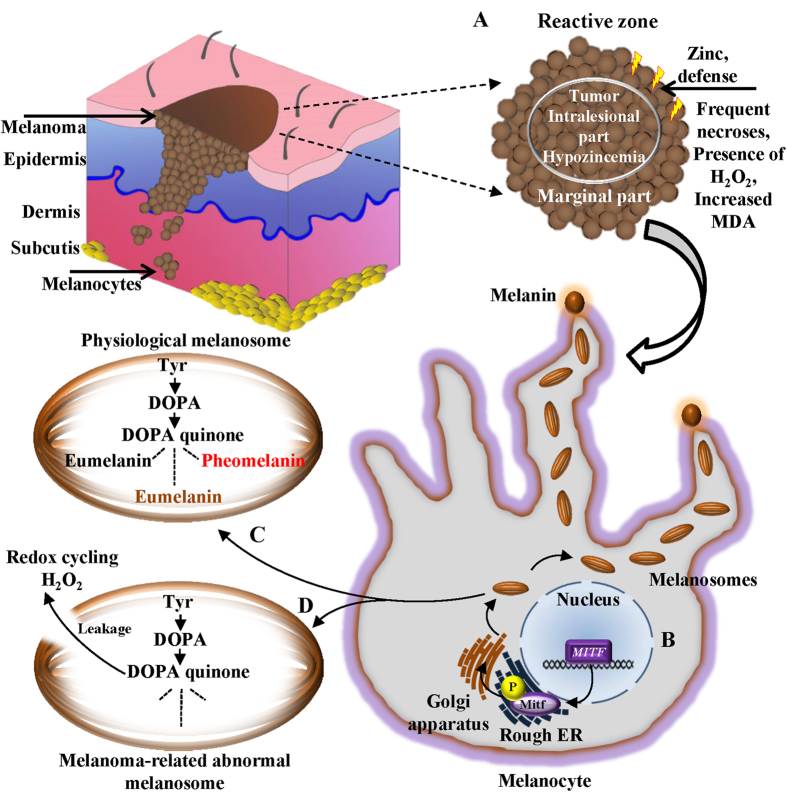
A schematic depiction of a plausible role of zinc in melanoma progression. Marginal parts of tumour border with a reactive zone of an adjacent healthy tissue. In these parts of melanoma, frequent necroses, a presence of H_2_O_2_ and elevation of malondialdehyde (MDA) can be found. (**A**) High zinc in marginal sections of melanoma may indicate a physiological defence of zinc-containing antioxidant molecules against the potential danger of ongoing oxidative stress. Although intralesional regions of melanomas generally exhibit hypozincemia status, oxidative stress in reactive zones/marginal parts interface can likely result in an oxidative stress-triggered transport of zinc-containing antioxidant molecules from adjacent healthy tissue and temporary accumulation of zinc. (**B**) Physiologically, an expression of melanosomes is regulated by microphthalmia-associated transcription factor gene (*MITF*) and phosphorylation (P) of the homonymous *MITF*-encoded protein (Mitf). (**C**) Translated melanosomes are the place for entire melanin synthesis, starting by action of tyrosinase, producing L-3,4-dihydroxyphenylalanine (DOPA) from tyrosine (Tyr). Tyrosinase further produces DOPA quinone from DOPA. DOPA quinone can be converted through a sequence of reactions to various types of melanins (black and brown eumelanins or red to yellow pheomelanin). (**D**) Noteworthy, another plausible reason for the free radicals formation in melanoma is a presence of abnormal and incomplete melanosomes, causing a significant leakage of the reactive melanin prescursors, causing oxidative stress in the pigmented tumours through redox cycling and an accumulation of zinc ions originating from antioxidant molecules from adjacent tissues as a kind of physiological protection.

Serum levels of Zn and Cu in melanoma patients have been suggested as valuable diagnostic and prognostic parameters but have yielded conflicting results. The Cu level (but not the Zn level) was generally elevated in melanoma patients, reflecting the degree and extent of tumour activity^26^. By contrast, serum Cu concentrations were identical in melanoma patients and healthy individuals, whereas the serum Zn concentration was significantly increased in melanoma patients^27^. In tissue sections, the Zn level was elevated in the majority of melanomas in comparison with the skin of healthy controls. However, the Cu level was increased in some melanoma patients^28^. The content of melanosomes in skin melanocytes is considerably lower than in melanoma cells, adequately explaining the results of the tissue analysis. Serum concentrations of Zn and Cu are probably greatly influenced by changes in melanosomal membrane permeability due to differences in the cellular milieu, with consequent leakage of reactive melanin precursors (mostly DOPA quinones and semiquinones) whose redox cycling reactions produce H_2_O_2_^29^. As a type of physiological defence, cells can over-express Zn-containing antioxidant molecules or transport and accumulate them from adjacent tissues. Another pivotal biometal, Cu, acts as a cofactor of tyrosinase, the principal enzyme in the synthesis of melanin pigment from tyrosine^30^. This process is schematically depicted in Fig. 7.

The suggested novel software-supported technique for Zn and Cu mapping permits not only simultaneous quantification in the same tissue cryosection but also detection of both metal ions in very small, histologically characterized zones of tissue, an important advantage compared to their determination in tissue homogenates or in whole tissue sections. We used skin melanoma samples from MeLiM animals of various ages to develop and validate this technique. Our findings (Fig. 6A–C) indicate that the Zn content of a given zone is approximately 3 or 4 times higher than that of Cu (Fig. 6D–F). Moreover, the content of both metals declines as a result of advancing spontaneous regression (due to destruction of melanoma cells by anti-tumour immune reaction). One may suggest that the reason for these changes should be somewhat connected with the expression of proteins, mainly the metal binding ones. From hundreds of the proteins able to bind Cu and Zn, we highlighted tyrosinase and Tyrp 2 (tyrosinase-related protein 2)/Dct (dopachrome tautomerase), two membrane-bound glycoproteins and key enzymes for the synthesis of melanin, where the mentioned metal ions serve as cofactors in the metal-binding sites^31^. Moreover, so called “universal soldiers” metallothioneins (MTs) also have binding capacities for Zn and Cu, whereas these are somewhat related to cancer related processes^32^,^33^,^34^. On the other hand, imaging metal species in biological tissue presents a complex analytical challenge: a suitable strategy requires a balance of sensitivity, selectivity and spatial resolution^35^. Hare *et al*. recently showed how they could increase spatial resolution of protein detection using gold-labelled immunohistochemical approach for LA-ICP-MS imaging tyrosine hydroxylase. Their results were associated with iron distribution, whereas findings are critical for further research in the field of neurotransmitters. In addition, our results suggest that the content of Zn and Cu for a given zone fluctuates during the postnatal period, mainly in the earliest ages (4–8 weeks of age). Ten weeks of age appears to be a turning point in the transition between tumour growth and spontaneous regression in MeLiM melanoma^5^. These relations need to be further related to protein spatial distribution, where the above mentioned enzymes and metallothioneins will be further targets.

## Material and Methods

### Animals and tissue samples

Ten MeLiM animals with multiple skin nodular melanomas (2 pigs each at ages of 4, 6, 8, 15 and 22 weeks) were used in this experiment. One melanoma was excised from each animal under total anaesthesia [premedication with intramuscular (i.m.) atropine 0.5 mg/minipig (Hoechst-Biotika, Slovak Republic), followed by Stresnil 1 mg/kg of body weight (Janssen Pharmaceutica N.V., Belgium) and Narcotan inhalation (Leciva, Czech Republic)]. Vetalgin (0.5 mg/kg of body weight; Intervet International, Germany) was applied i.m. to control pain after tumour excision and wound suturing. This experimental treatment was performed in accordance with the Project of Experiment approved by the Animal Science Committee of the IAPG AS CR, v.v.i. (Libechov, Czech Republic), following the rules of the European Convention for the Care and Use of Laboratory Animals.

Tissue blocks (approximately 8 × 5 mm) of irregular (trapezoidal) shape were obtained from each melanoma. The specific polygonal shape of the cross-section of each block facilitates identical orientation of serial tissue sections used for both histology and LA-ICP-MS. Tissue blocks were placed on a piece of cork, covered with Jung Tissue Freezing Medium (Leica Micro-systems, Germany), and frozen in liquid nitrogen immediately after tumour excision. Serial cryosections (8 μm and 30 μm thick for histology and for LA-ICP-MS, respectively) were prepared by the Leica CM 1850-Cryostat (Leica, Germany), air dried (30 min) and stored at −20 °C until further processing.

Prior to this study, we did a preliminary one, where we tested two issues. The first one was whether we are capable of measuring spatial distribution of metal ions within a tissue section. For this purposes, we prepared sections of various thickness (from 5 to 50 μm) and found 30 μm the best from the point of view of sensitivity and also not influencing variability of a spatial distribution within a tumour.

### Histology

Haematoxylin-eosin staining was applied to observe tissue structure. Cryosections (8 μm thickness) were fixed with ethanol (20 min), washed with distilled water (3 times, 5 min each) and treated with Weigert’s haematoxylin (20 min) for nuclei staining. Then, the cryosections were washed with running tap water (20 min), followed by distilled water (3 times, 5 min each). The cytoplasm was counterstained with 1% eosin alcoholic solution (1 min). After washing with distilled water (3 times, 5 min each), the stained sections were embedded in glycerine jelly. Whole cryosections were scanned by a VS120 Olympus microscope with OlyVIA software (Olympus, Japan). GIMP software was used to identify four histologically different zones of tissue in the scanned pictures. Three to six rectangular areas of each zone per cryosection were chosen for comparison with Zn and Cu maps to detect their local contents.

### Elemental mapping procedure with LA-ICP-MS

Imaging experiments were performed using an LA-ICP-MS setup consisting of the laser ablation system UP213 (Supercell©, NewWave, USA) operated at a wavelength of 213 nm. The system was equipped with a CCD camera for investigation of the sample during laser ablation. The ablated material was washed away using helium (1.0 l/min) from the ablation chamber (Supercell©). Argon flow (0.6 l/min) was admixed into a flow of helium with the sample aerosol behind the ablation cell. Hence, the total gas flow was 1.6 l/min. This mixture was fed into a quadrupole ICP-MS spectrometer Agilent 7500CE (Agilent, Japan) equipped with a collision-reaction cell (CRC) for suppressing possible polyatomic interferences. The CRC was utilized in collision mode with a He (99.999%) flow rate of 2 ml/min. The integration time of monitoring was set to 0.1 s for the isotopes ^28^Si, ^63^Cu, ^66^Zn and 0.05 s for ^12^C, yielding a total sampling time of 0.43 s. The measured elements were chosen based on the analysed material (C as a matrix element of biological tissue and Zn as the elements studied in melanoma) and the microscope slide on which the thin section was deposited (silicon as a glass matrix element). The silicon signal emerged immediately once the laser beam reached the tissue/glass interface, thus indicating complete sampling of a tissue layer. The selection of the ablation parameters is explained in Results and Discussion.

### Post-processing of collected digital data and statistical analysis

The above-mentioned techniques generated pairs of digital images that were approximately similar but not sufficiently similar for exact indexing among images. These data were submitted to the procedure described by the flow chart in Fig. 1. The registration of the data is described below, and the statistical evaluation based on the mixed effect model is detailed in Supplementary Note 1. To define corresponding zones, the relationship between the registered images was used to map the selected areas identified in the histologically stained cryosections onto the respective maps of Zn, Cu and C (Fig. 3). The distribution of metal contents in the corresponding zones was compared and further explored by means of the mixed effect model described in Supplementary Note 1.

### Reproducible analysis of the data

The methods mentioned in the sections on the image registration of the considered images and content maps as well as on the statistical analysis–are provided for the purposes of re-use and reproducible analysis. In the case of image registration, which was performed in the MATLAB programing language, the method is provided in the form of scripts (m-files). The scripts and the necessary data (images and concentration maps) are wrapped in an archive and are designed to simplify the registration process to the execution of only a few scripts. The scripts may be executed by the MATLAB programing language as well as the open source alternative Octave.

The statistical analysis, which was performed using the statistical software R, is provided in a similar form, i.e., as an R script.

### Image registration

The archive containing the scripts for the image registration can be downloaded from Dryad. The implementation relies on the Image processing toolbox and the Optimization toolbox in the MATLAB programming language. However, the image registration may be easily performed using the Octave program instead of MATLAB. The image registration is performed by ***register_all_at_once**.**m*** script. The script steps through folders containing the images to be registered and performs the registration between the silhouette of the histological scan and the silhouette of the image of the sample to be ablated and measured as well as between the silhouette of the image of the sample to be ablated and measured and the silhouette of the measurements resulting from LA-ICP-MS. The images required for the registration are ***hist_si**.**png***, the silhouette of the histological scan; ***abla_si**.**png***, the silhouette of the image of the sample before ablation; and ***matr_si**.**png***, the silhouette of the concentration map. The results of the script are the transformation matrices stored in the sample folders under the name ***trans**.**mat*** and diagnostic images indicating the quality of the registration in the form of differences.

The resulting transformation matrices may be used for direct indexing among images and content maps. For example, the estimation of average metal contents in annotated spots may be performed by the script ***means_by_spots**.**m***. Similar to the ***register_all_at_once**.**m*** script, the program steps through the sample folders and estimates average contents in spots annotated in image ***anot**.**png***. The information about the spots is extracted from the image in file ***spots**.**mat***. The result is a csv file. Files for all samples may be combined and used for statistical analysis.

### Statistical analysis

Statistical analysis was performed in R statistical software and is presented as a script. The case bootstrap test relies on the output of a random number generator. To ensure the reproducibility of the results, the state of the random number generator is fixed. The analyses in the script ***lmer_analyses**.**R*** sequentially tests the two hypotheses on the Zn and Cu meta-concentrations stored in the file MeLiM_metals_data.csv, namely “The average Zn (or Cu) content in the GMT zones is lower than that in the remaining zones (ESR, LSR and FT)”. The analysis is very computationally intensive; the execution of the script may last a few hours. The results of the analysis are the fitted linear mixed effect models and the ***p*** values estimated by the case bootstrap test.

## Additional Information

**How to cite this article:** Anyz, J. *et al*. Spatial mapping of metals in tissue-sections using combination of mass-spectrometry and histology through image registration. *Sci. Rep.* **7**, 40169; doi: 10.1038/srep40169 (2017).

**Publisher's note:** Springer Nature remains neutral with regard to jurisdictional claims in published maps and institutional affiliations.

## Supplementary Material

Supplementary Data R1

## Figures and Tables

**Table 1:** The averages and differences of Zn and Cu contents as estimated by the linear mixed effect model and corrected by random effects are provided in the relevant section of supplementary; the diagonal values are the estimated average contents in the respective zones, and the off-diagonal values indicate their differences.

	Coefficients	GMT	SR + FT	*p* values	GMT	SR + FT
Zn	GMT	495.90	−146.38	GMT	1.00000	0.00307
	SR + FT	146.38	349.52	SR + FT	0.99693	1.00000
Cu	GMT	126.26	−44.80	GMT	1.00000	0.04277
	SR + FT	44.80	81.46	SR + FT	0.95723	1.00000
