# Supplementary material for: Spatial mapping of metals in tissue-sections using combination of mass-spectrometry and histology through image registration
Source: Sci Rep. 2017 Jan 10;7:40169. doi: 10.1038/srep40169 (PMC5223221; doi:10.1038/srep40169)
Supplement: Supplementary Data R1 [file srep40169-s1.doc]

**Spatial mapping of metals in tissue-sections using combination of mass-spectrometry and histology through image registration**

Jiri Anyz^1^, Lenka Vyslouzilova^2^, Tomas Vaculovic^3,4^, Michaela Tvrdonova^3,4^, Viktor Kanicky^3,4^, Hajo Haase^5^, Vratislav Horak^6^, Olga Stepankova^1,2^, Zbynek Heger^7,8^, Vojtech Adam^7,8*^

*^1^Department of Cybernetics, Faculty of Electrical Engineering, Czech Technical University, Technicka 2, CZ-166 27 Prague 6, Czech Republic*

*^2^Czech Institute for Cybernetics and Informatics, Czech Technical University, Technicka 2, CZ-166 27 Prague 6, Czech Republic*

*^3^Department of Chemistry, Faculty of Science, Masaryk University, Kotlarska 2, CZ-611 37 Brno, Czech Republic*

*^4^Central European Institute of Technology, Masaryk University, Kamenice 5, CZ-625 00 Brno, Czech Republic*

*^5^Department of Food Chemistry and Toxicology, Berlin Institute of Technology, Gustav-Meyer-Allee 25, D-133 55 Berlin, Germany*

*^6^Laboratory of Tumour Biology, Institute of Animal Physiology and Genetics, Academy of Sciences of the Czech Republic, v.v.i., Rumburska 89, CZ-277 21 Libechov, Czech Republic*

*^7^Department of Chemistry and Biochemistry, Mendel University in Brno, Zemedelska 1, CZ-613 00 Brno, Czech Republic*

*^8^Central European Institute of Technology, Brno University of Technology, Technicka 3058/10, CZ-616 00 Brno, Czech Republic*

***Corresponding author**

Vojtech Adam, Department of Chemistry and Biochemistry, Mendel University in Brno, Zemedelska 1, CZ-613 00 Brno, Czech Republic; E-mail: vojtech.adam@mendelu.cz; Phone: +420-5-4513-3350; Fax: +420-5-4521-2044.

**Supplementary Note 1**

*Statistical analysis*

*The output of the analysis with biological significance*

The preceding procedures provided us with the data for 10 tissue samples each from 10 individual animals of five postnatal ages. Three histologically different zones were observed in each sample taken from the minipigs at the ages of 6 weeks or more (GMT, ESR and LSR at the age of 6 weeks; and ESR, LSR and FT at the ages of 15 and 22 weeks), whereas only two zones (GMT and ESR) were detected in the samples from the 4-week-old minipigs. Approximately 5 spots of each zone per individual sample were annotated, yielding 125 annotated spots in total. In each spot, the contents of the considered metals were determined, and the resulting values were subjected to statistical analysis. The sample mean averages of Zn and Cu content obtained by transforming the histological annotation into the content map coordinates exhibited a tendency to decrease during spontaneous disintegration of the melanoma tissue and its rebuilding into fibrous tissue (see **Supplementary Tab. 1**). However, the presence of many outliers in the detailed review of values as they occur (e.g. in **Figure 3a.** and **3f.**) indicates that the individual animals had different overall metal contents (in the whole tissue sample), which is why the mixed effect model with random intercepts was used.

**Supplementary Tab. 1A**: The averages and standard deviations of Zn content according to samples and histological zones. The samples are ordered according to the age of the animals from youngest (N125, N129) to oldest (L618, L619). The right column presents the averages of Zn content in the samples, irrespective of the histological zones. The bottom row presents the averages and standard deviations of Zn content in the histological zones, irrespective of sample information.

|  | **Age** | **GMT** | **ESR** | **LSR** | **FT** | **Sample** |
| --- | --- | --- | --- | --- | --- | --- |
| **N125** | 4 | 483.3 (72.4) | 415.9 (100.2) | *NA* | *NA* | 460.9 (79.8) |
| **N129** | 4 | 542.4 (230.4) | 504.0 (148.0) | *NA* | *NA* | 523.2 (180.4) |
| **N113** | 6 | 545.5 (*NA*) | 264.1 (48.6) | 253.6 (34.0) | *NA* | 283.2 (91.3) |
| **N115** | 6 | 270.4 (113.1) | 285.3 (90.3) | 215.8 (28.6) | *NA* | 255.1 (78.8) |
| **N92** | 8 | 893.6 (192.9) | 659.8 (220.7) | 699.1 (152.2) | *NA* | 769.5 (217.2) |
| **N93** | 8 | 499.6 (59.0) | 421.6 (141.3) | 235.6 (134.5) | *NA* | 392.7 (156.1) |
| **L669** | 15 | *NA* | 187.9 (57.5) | 190.8 (83.1) | 203.3 (65.7) | 193.3 (64.7) |
| **L670** | 15 | *NA* | 321.0 (218.7) | 341.3 (60.6) | 747.4 (*NA*) | 377.4 (196.6) |
| **L618** | 22 | *NA* | 145.5 (18.4) | 145.9 (40.4) | 143.9 (39.2) | 145.2 (31.6) |
| **L619** | 22 | *NA* | 423.0 (143.8) | 475.6 (81.3) | 389.9 105.1) | 433.0 (106.4) |
| **Zone** | | 588.5 (249.9) | 355.6 (200.0) | 303.7 (174.1) | 288.7 (179.0) |  |

The differences were estimated as fixed effects in the prediction of the metal contents according to the tissue type indicator. The indicator of the animal was utilized as a random intercept. The model for meta-concentration may be described by the following equation:


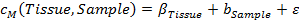


where the term
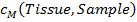
 denotes the response metal content (Zn or Cu) depending on the tissue type indicator and the sample indicator. ***β_Tissue_*** is the fixed effect coefficient corresponding to the ***Tissue*** type; ***b_Sample_*** is the random effect coefficient for the considered ***Sample***; and ***ε*** is the error term. Because the predictors are both indicator variables, ***β_tissue_*** and ***b_sample_*** coefficients are in fact the coefficients for each level of the indicator variable. The random intercepts ***b*** are assumed to be drawn from a normal distribution with a zero mean and act as compensation for the various overall metal contents in the samples. In this setting, the random effect coefficients do not alter the actual differences among tissue types. The average contents of Zn and Cu in histologically different zones are summarized in **Supplementary Tab. 1**. The values in the last row of **Supplementary Tab. 1A,** which present the average contents for the different tissue zones, reveal the tendencies to decrease, whereas the last column, which presents the average content among all annotated spots in a certain sample, reveals substantial variation among samples.

The exploration of the values in **Supplementary Tab. 1B** led to the formulation of two hypotheses to be tested specifically on the available data. The first hypothesis **H1** claims, “The metal content in growing melanoma tissue is smaller than or equal to the concentration in the remaining tissue zones, i.e., in the tissue undergoing spontaneous regression (early and late) or in the fibrous tissue.” The second hypothesis is a minor modification of the first hypothesis; hypothesis **H2** claims, “The metal content in the zones of growing melanoma tissue and early spontaneous regression is lower than or equal to that in the zones of late spontaneous regression and/or the fibrous tissue.” To maintain an overall error rate of 0.05, the threshold for ***p*** values (used in the decision for whether the hypotheses can be refuted) is 0.0125 according to the Bonferroni correction for multiple comparison. The estimated characteristics and respective ***p*** values resulting from non/parametric bootstrap tests of the considered hypotheses are shown in **Supplementary Tab. 2**.

**Supplementary Tab. 1B**: The averages and standard deviations of Cu content according to samples and histological zones. The samples are ordered according to the age of the animals from youngest (N125, N129) to oldest (L618, L619). The right column presents the averages of Cu content values in the samples, irrespective of the histological zones. The bottom row presents the averages and standard deviations of Cu content in the histological zones, irrespective of sample information.

|  | **Age** | **GMT** | **ESR** | **LSR** | **FT** | **Sample** |
| --- | --- | --- | --- | --- | --- | --- |
| **N125** | 4 | 82.7 (10.1) | 73.6 (5.5) | *NA* | *NA* | 79.7 (9.5) |
| **N129** | 4 | 67.5 (6.5) | 72.5 (15.1) | *NA* | *NA* | 70.0 (11.1) |
| **N113** | 6 | 1348.0 (*NA*) | 295.6 (432.6) | 96.9 (17.2) | *NA* | 300.5 (451.4) |
| **N115** | 6 | 116.9 (42.2) | 123.8 (31.7) | 103.5 (25.5) | *NA* | 114.4 (30.6) |
| **N92** | 8 | 119.3 (36.4) | 160.5 (45.5) | 151.8 (10.5) | *NA* | 140.8 (40.4) |
| **N93** | 8 | 72.6 (9.5) | 65.1 (10.7) | 60.9 (10.6) | *NA* | 66.6 (10.8) |
| **L669** | 15 | *NA* | 53.9 (10.7) | 57.1 (22.8) | 57.2 (17.9) | 56.0 (16.5) |
| **L670** | 15 | *NA* | 95.5 (29.1) | 104.9 (15.9) | 129.9 (*NA*) | 103.5 (23.1) |
| **L618** | 22 | *NA* | 100.0 (32.6) | 86.0 (25.6) | 66.8 (19.4) | 85.3 (28.7) |
| **L619** | 22 | *NA* | 62.5 (10.9) | 55.5 (12.4) | 65.0 (12.6) | 60.5 (12.2) |
| **Zone** | | 142.8 (253.1) | 119.6 (163.1) | 84.3 (32.7) | 67.7 (22.5) |  |

**Table 2.** The average differences of Zn content between the GMT zone and the rest of the tissue zones. The values on the diagonal in the left half of the table denote the average content of Zn in the examined histological zones, and the off-diagonal values are the estimated differences between the averages of Zn content in the histological zones. The values in the right half of the table indicate the ***p*** values resulting from the case bootstrap test for hypothesis H1 or H2.

**Supplementary Tab. 2A:** Comparison of Zn content according to hypothesis H1.

| **Coefficients** | GMT | SR + FT | ***p* values** | GMT | SR + FT |
| --- | --- | --- | --- | --- | --- |
| GMT | 495.90 | -146.38 | GMT | 0.00001 | 0.00307 |
| SR + FT | 146.38 | 349.52 | SR + FT | 0.99693 | 0.00001 |

**Supplementary Tab. 2B:** Comparison of Zn content according to hypothesis H2.

| **Coefficients** | GMT + ESR | LSR + FT | ***p* values** | GMT + ESR | SR + FT |
| --- | --- | --- | --- | --- | --- |
| GMT + ESR | 401.84 | -48.49 | GMT + ESR | 0.0001 | 0.1145 |
| SR + FT | 48.49 | 383.35 | SR + FT | 0,8855 | 0.0001 |

**Supplementary Tab. 2C:** Comparison of Cu content according to hypothesis H1.

| **Coefficients** | GMT | SR + FT | ***p* values** | GMT | SR + FT |
| --- | --- | --- | --- | --- | --- |
| GMT | 151.70 | -55.81 | GMT | 0.0001 | 0.0642 |
| SR + FT | 55.81 | 95.89 | SR + FT | 0.9358 | 0.0001 |

**¨**

**Supplementary Tab. 2D:** Comparison of Cu content according to hypothesis H2.

| **Coefficients** | GMT + ESR | LSR + FT | ***p* values** | GMT + ESR | LSR + FT |
| --- | --- | --- | --- | --- | --- |
| GMT + ESR | 126.26 | -44.80 | GMT + ESR | 0.00001 | 0.04277 |
| LSR + FT | 44.80 | 81.46 | LSR + FT | 0.95723 | 0.00001 |

Only the ***p*** value for the comparison of Zn content indicated in **Supplementary Tabs. 2A-D** is lower than the threshold value of 0.0125, and consequently, hypothesis **H1** can be refuted. Although our data confirm that “the content of Zn in the zone of growing melanoma tissue (GMT) is significantly greater than in all remaining zones, which represent consecutive stages of the tumour tissue arising as a result of the spontaneous regression of melanoma (ESR, LSR) and its final rebuilding into fibrous tissue,” they do not provide sufficient evidence to refute hypothesis **H1** for Cu and hypothesis **H2** for either metal of interest.
